# Assessing supply-side barriers to uptake of intermittent preventive treatment for malaria in pregnancy: a qualitative study and document and record review in two regions of Uganda

**DOI:** 10.1186/s12936-016-1405-4

**Published:** 2016-07-04

**Authors:** Christian Rassi, Kirstie Graham, Patrobas Mufubenga, Rebecca King, Joslyn Meier, Sam Siduda Gudoi

**Affiliations:** Malaria Consortium, Development House, 56-64 Leonard Street, London, UK; Malaria Consortium Uganda and PAMU Consults (U) Ltd, Plot 577, Block 15, Nsambya Road, Kampala, Uganda; Nuffield Centre for International Health and Development, Leeds Institute of Health Sciences, University of Leeds, Leeds, UK; Malaria Consortium Uganda, Plot 25 Upper Naguru East Road, Kampala, Uganda

**Keywords:** Malaria in pregnancy, Prevention, IPTp, Antenatal care, ANC, COMDIS-HSD, Malaria Consortium

## Abstract

**Background:**

Intermittent preventive treatment in pregnancy (IPTp) with sulfadoxine–pyrimethamine (SP), provided as part of routine antenatal care (ANC), is one of three malaria-in-pregnancy prevention and control mechanisms recommended by the World Health Organization (WHO). However, despite high ANC attendance and increased efforts to address known obstacles, IPTp uptake figures have remained low. This study aimed to identify and assess barriers that continue to impede IPTp uptake in Uganda, in particular for women who attend ANC. The paper focuses on supply-side barriers, i.e., challenges relating to the health service provider.

**Methods:**

In-depth interviews were conducted in two regions of Uganda in November 2013 and April/May 2014 with four different target audiences: seven district health officials, 15 health workers, 19 women who had attended ANC, and five opinion leaders. In addition, a document and record review was carried out at four health facilities.

**Results:**

Guidelines with regard to IPTp provision in Uganda have been shown to be inconsistent and, at the time of the research, did not reflect the most recent WHO policy recommendation. There is a lack of training and supervision opportunities for health workers, resulting in poor knowledge of IPTp guidelines and uncertainty about the safety and efficacy of SP. ANC is not consistently offered in health facilities, leading to some women being denied services. While strengthening of the supply chain appears to have reduced the occurrence of stock-outs of SP in public facilities, stock-outs reportedly continue to occur in the private sector. There are also sources of data inaccuracy along the data recording and reporting chain, limiting policy makers’ ability to react adequately to trends and challenges.

**Conclusions:**

Given the high ANC attendance rates in Uganda, supply-side barriers are likely to account for many missed opportunities for the provision of IPTp in Uganda. Improvements will require consistent provision of ANC, implementation of current WHO IPTp policy recommendations, supply of SP to the private sector, availability of clear guidelines, as well as improved training and supervision for health workers. Improving facility and district-level recording and reporting will further strengthen the country’s ability to address uptake of IPTp.

**Electronic supplementary material:**

The online version of this article (doi:10.1186/s12936-016-1405-4) contains supplementary material, which is available to authorized users.

## Background

Malaria in pregnancy can cause serious harm to the health and well-being of both mother and child. It can lead to maternal anaemia, which is estimated to contribute to between 10 and 25 % of all maternal deaths in endemic areas [1]. Malaria in pregnancy also increases the risk of miscarriage and intra-uterine growth restriction, as well as pre-term and low birth weight delivery [2]. Each year, 27.6 million pregnancies leading to live births occur in malaria-endemic areas in Africa and it is estimated that, in the absence of preventive interventions, 12.4 million of them would have been exposed to malaria infection [3].

The World Health Organization (WHO) recommends three strategies for the prevention and control of malaria in pregnancy: (i) effective case management for malaria illness and anaemia; (ii) use of insecticide-treated nets; and, (iii) intermittent preventive treatment in pregnancy (IPTp) with sulfadoxine–pyrimethamine (SP) [4].

IPTp involves the administration of curative doses of an anti-malarial drug to pregnant women residing in areas of moderate or high transmission in Africa, typically as part of the focused antenatal care (ANC) package. While SP is no longer recommended for the treatment of confirmed clinical cases of malaria, the drug is still considered the most efficacious anti-malarial for preventive use in pregnant women [5].

Previously, WHO guidelines recommended at least two doses of SP after quickening at the second and third scheduled ANC visit [4]. Since 2012, WHO recommends IPTp with SP for all pregnant women at each scheduled ANC visit, except during the first trimester and provided that doses are given 1 month apart. IPTp should be administered as directly observed therapy (DOT). Pregnant women who are human immunodeficiency virus (HIV)-positive and who receive co-trimoxazole prophylaxis should not be given IPTp [5].

Despite high use of ANC services in many countries of sub-Saharan Africa, in 2014 only 40 % of women in 36 reporting countries received two doses of IPTp and 17 % received three or more doses [6], suggesting that a large number of opportunities to provide IPTp during ANC continue to be missed. A broad range of obstacles to the effective provision of ANC and IPTp have been discussed in the literature [7, 8], including issues relating to leadership and coordination, human resources, supply chain, and service delivery.

This study sought to identify and assess barriers that continue to impede uptake of IPTp in Uganda, despite high ANC uptake and increased efforts by government and implementation partners to address some of the commonly described bottlenecks. Using qualitative methods, this research attempted to gain an in-depth understanding of how key informants (district health officials, health workers, opinion leaders, pregnant women, and mothers) perceive the provision of IPTp and how this could affect its uptake, in particular for those women who do attend ANC. Accuracy and quality of available facility and district-level data were explored through a review of documents and records relating to ANC and IPTp. The research was intended to inform the development of a pilot intervention to increase uptake of IPTp.

This paper focuses on key challenges identified relating to supply-side issues, i.e., those challenges relating to the health service provider, including barriers relating to accessibility due to availability of services, affordability due to service charges, and acceptability from health workers’ point of view [9]. The findings of the document and record review are also reported here, as accuracy and quality of available coverage data will affect policy makers’ and service providers’ ability to gauge the extent of the problem and react appropriately to challenges. Demand-side barriers will be presented in a separate paper.

## Methods

### Study design

The study involved qualitative elements and a document and record review. Reporting of the qualitative study methods follows the consolidated criteria for reporting qualitative research (COREQ) [10].

### Study setting

#### ANC and IPTp in Uganda

All of Uganda’s population is thought to live in areas of high malaria transmission [6]. More than 600,000 pregnancies are at risk from malaria infection each year [3]. Countrywide IPTp programme implementation in Uganda started in 2001 [11]. As in most countries, the treatment is provided to pregnant women as part of routine ANC visits. The various IPTp guidelines and policies in use in Uganda have been found to be “inconsistent or unclear” [12] and, at the time of the research, did not yet reflect the latest WHO policy recommendation of monthly administration from the second trimester, which was only formally adopted in May 2016.

According to Demographic and Health Survey data, coverage of at least two doses of IPTp was 25 % in 2011 [13], down from 32 % in 2009 [14]. Approximately 90 % of women visited ANC at least twice [13]. Recognizing the challenge, government and implementation partners have stepped up efforts to increase uptake of IPTp. However, coverage gaps remain.

#### Study regions and districts

**Fig. 1:**
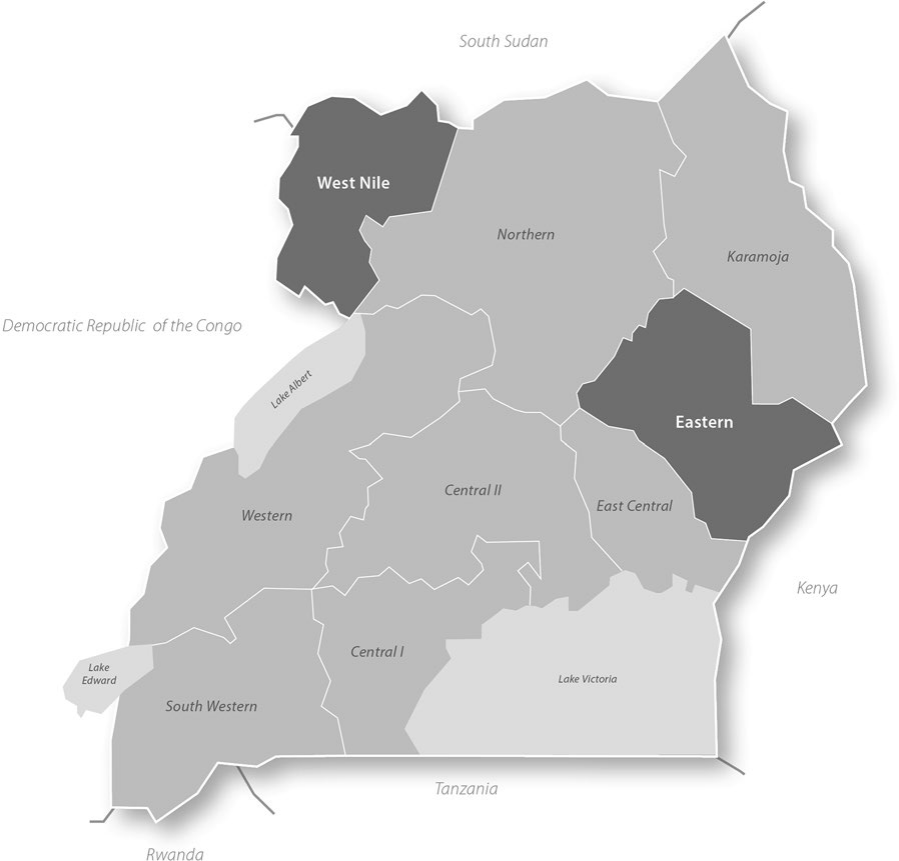
Map of the Republic of Uganda. Study regions highlighted in *dark grey*

Taking into account feasibility with regard to time and budget, two regions of Uganda were selected, Eastern and West Nile (see Fig. 1), which represent a broad range of the country’s geographical, linguistic and ethnic diversity. The two regions were also selected because conducting the study in those regions was expected to provide an insight into barriers to and enablers of IPTp uptake, as, according to the most recent Uganda Demographic Health Survey for which data are available, they were the regions reporting the highest and lowest uptake of IPTp. In Eastern, 36 % of women reported having received at least two doses of IPTp, while this figure was only 21 % in West Nile [13]. This may be a reflection of the fact that Eastern has benefited from several malaria prevention and control programmes, whereas West Nile is among those regions that have historically received lower levels of implementation partner support.

Within each region, one predominantly urban and one predominantly rural district were purposively selected for inclusion in the study sample. Table 1 presents an overview of IPTp and ANC uptake figures for the 2 years preceding data collection (2011 and 2012) from the study districts and regions. The data were extracted from the Republic of Uganda’s Health Management Information System (HMIS). It is noticeable that the pattern of high uptake of IPTp in Eastern and low uptake in West Nile found by household surveys is not consistently reflected in the HMIS data, which are based on reports from health facilities. Note, however, that an assessment of health facility data quality in Uganda carried out by WHO in 2011 concluded that “intervention coverage estimates are often poor” [15]. This conclusion is supported by the results of the document and record review carried out as part of this study. The study team did not have access to HMIS data at the time of selecting study regions and districts.

**Table 1:** Antenatal care and IPTp uptake data (%) in study districts, 2012–2013 (Source: Health Management Information System, Republic of Uganda)

	ANC1^a^		ANC4^b^		IPT1^c^		IPT2^d^	
	2011	2012	2011	2012	2011	2012	2011	2012
Uganda	90	84	29	28	58	79	44	50
Eastern	73	67	18	18	79	83	51	52
Urban study district	57	66	15	29	97	96	70	76
Rural study district	43	61	6	9	78	93	66	55
West Nile	46	57	18	22	83	86	59	62
Urban study district	61	101	19	36	76	81	50	54
Rural study district	20	22	13	11	92	92	71	69

^a^ANC1: First antenatal care visit; denominator: expected pregnancies (5 % of total population in the area)

^b^ANC4: Fourth antenatal care visit; denominator: expected pregnancies (5 % of total population in the area)

^c^IPT1: First dose of IPTp; denominator: ANC1

^d^IPT2: Second dose of IPTp; denominator: ANC1

#### Health facilities

In each of the four study districts, two health facilities were selected, representing all levels of health centres (II, III, IV and hospital) that provide ANC services in Uganda. Only public and private not-for-profit (PNFP) facilities were selected, as those types of facilities were expected to experience similar challenges. The Ministry of Health does not provide specific support on IPTp to private for-profit health facilities and this type of facility was therefore excluded from the sample.

**Fig. 2:**
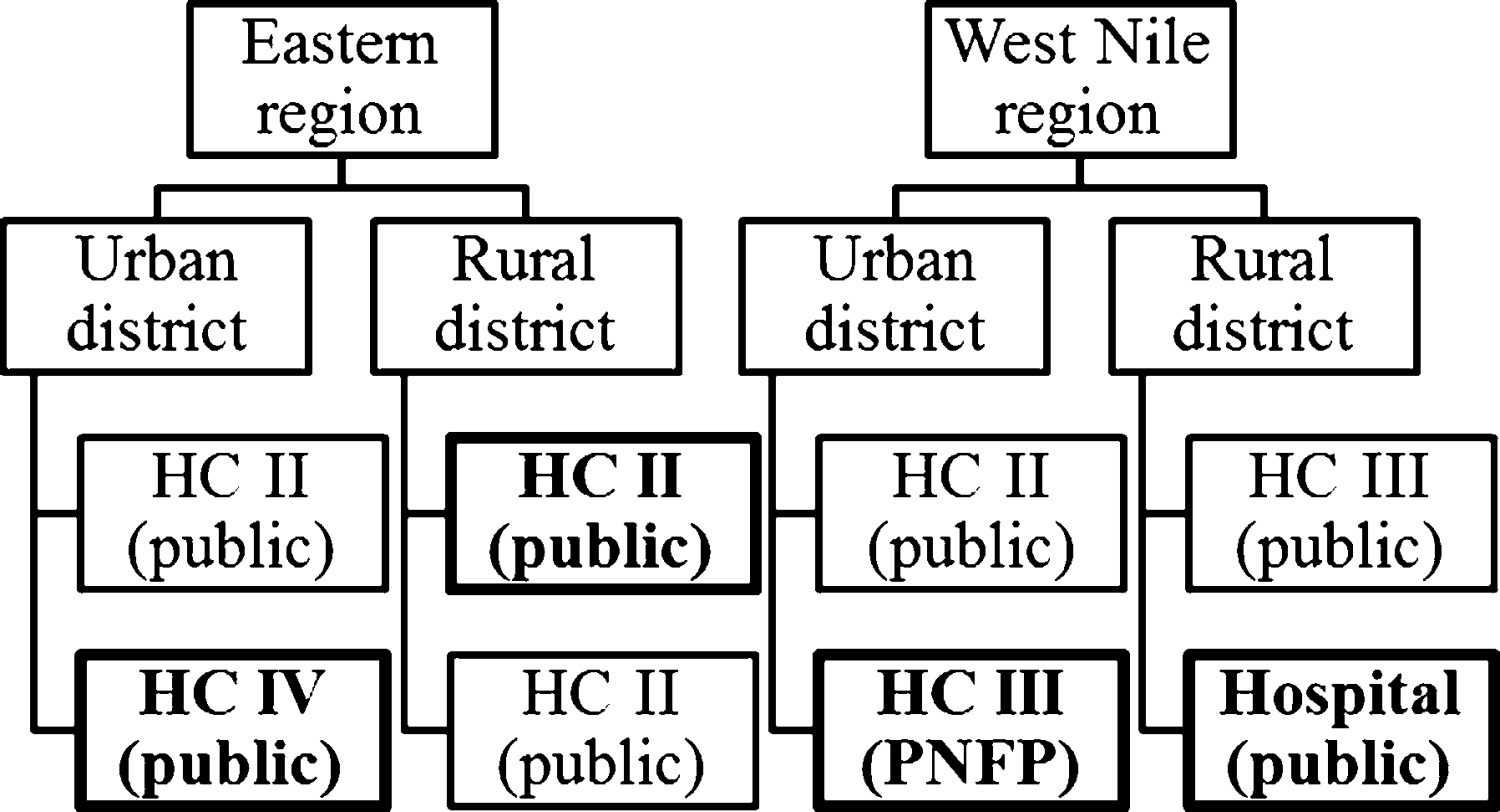
Type of health facilities visited per study district. *HC* health centre, *PNFP* private not-for-profit. Health facilities selected for the document and record review have been highlighted in *bold*

Health facilities were selected based on convenience, but ensuring that the respective number of types of facilities included in the sample approximately corresponded to the proportion of this type of facility in the country [16]. One of the two health facilities sampled in each district was purposively selected for the document and record review, again ensuring a mix of different types of health facilities were represented. Originally, two PNFP facilities had been selected in line with this type of facility’s overall proportion among health facilities in the country. However, one of the PNFP facilities originally selected, a health centre II, was found to be no longer operational shortly before the research team’s planned visit and an alternative public health centre II in the same district was selected at short notice. See Fig. 2 for an overview of the types of health facilities selected per study district.

### Participant selection

A total of 46 in-depth interviews were carried out. None of the selected interviewees who were approached by the field team refused to be interviewed. Table 2 presents an overview of in-depth interviews conducted per type of respondent, as well as how interviewees were approached and where the interviews took place. Taking into account available time and budget and recognizing the systematic selection of study regions, districts, health facilities, and participants, this sample size was considered sufficient to adequately explore the research questions and to arrive at valid conclusions and recommendations.

**Table 2:** In-depth interviews conducted per type of respondent

Type of respondent	Number of respondents	Approached by	Location of interview
District health officials			
Malaria Focal Persons	3	District health officer, 1 week prior to research team’s visit to district	Respondents’ usual place of work
Assistant District Health Officer (Maternal and Child Health)	1		
Health Educator	1		
Health Management Information System Focal Person	1		
Stores Assistant	1		
Total district health officials	7		
Health workers			
In-charges	7	Facility in-charge, 1 week prior to research team’s visit to facility	Respondents’ usual place of work
Midwives	8		
Total health workers	15		
Women			
Pregnant and received IPTp	8	Researcher on day of visit to community	Respondents’ home or health facility
Recently given birth and received IPTp	8		
Recently given birth and did not receive IPTp	3		
Total women	19		
Opinion leaders			
Traditional birth attendants	2	Researcher on day of visit to community	Respondents’ home
Community councillor	1		
Teacher	1		
Retired midwife	1		
Total opinion leaders	5		

#### District health officials

Two district health officials per district were selected in consultation with the District Health Officer, based on their involvement in malaria in pregnancy planning, implementation and reporting. A total of seven interviews were conducted, as only one suitable district official was available in one district in West Nile during data collection. Interviewees at the district level included Malaria Focal Persons, as well as staff in charge of maternal and newborn health, supply chain management, health education, and HMIS data.

#### Health workers

At each health facility, two health workers were selected in consultation with the facility in-charge based on their involvement in provision and oversight of ANC services. One was generally the acting facility in-charge and the other was a midwife. In one facility in West Nile only one potential interviewee was available on the day the field team visited; hence, a total of 15 health workers were interviewed.

#### Women

During a first round of data collection, pregnant women or women who had given birth during the previous 12 months and who had attended ANC at least once were selected from health facilities’ ANC registers with the help of a health worker. Two women were selected per facility, one who was still pregnant and one who had recently given birth. Where possible, the team selected one woman who lived within walking distance from the facility and one woman from the edge of the facility’s catchment area. However, it was difficult to apply this criterion in the field and information about distance from the health facility was not captured consistently. A total of 16 women were interviewed initially.

During analysis of the data from the first round of data collection, it emerged that all women interviewed were frequent ANC attenders and held largely positive views of IPTp. It was felt that health workers’ involvement in identifying suitable interviewees may have biased the data collected. To obtain a more comprehensive understanding of women’s perceptions, a second round of data collection was carried out specifically to interview women who had attended ANC, but had not received a full course of IPTp despite being eligible at the time of the ANC visit. Fifteen women were therefore identified from facilities’ ANC registers by the field researchers without any involvement from health workers. Although the team intended to carry out four additional interviews, only three of these 15 women fit the inclusion criteria and were subsequently interviewed. All three had recently given birth. The remaining 12 women either reported that they had in fact received IPTp or that they had not been eligible to receive IPTp at the time of the ANC visit in question.

#### Opinion leaders

During the second round of data collection, the team also conducted five additional in-depth interviews with opinion leaders who were considered influential in shaping communities’ perceptions and practices with regard to ANC and IPTp. Opinion leaders were selected in consultation with the sub-county Secretary for Health and included traditional birth attendants, a community councillor, a teacher, and a retired midwife.

### Data collection

#### Field work

The first round of qualitative data collection was conducted during November 2013 by CR and eight field researchers. Five of the field researchers were female, three were male; all were university or college graduates with some previous field research experience and relevant local language skills. Prior to data collection, field researchers attended a 5-day training course on malaria in pregnancy and qualitative research techniques, which was conducted by CR. The second round of data collection was carried out over a period of 8 days in April and May 2014 by CR and three of the field researchers who had been involved in the first round of interviews. Only two out of the original four study districts were visited for the additional interviews: the urban district in Eastern region and the rural district in West Nile. Due to constraints in terms of time and logistics, this was considered sufficient as results from the first round of data collection did not differ widely between urban and rural districts within the same region.

Interviews lasted between 45 and 75 min. District health officials, health workers and opinion leaders were interviewed in English, while interviews with pregnant women and mothers were conducted in the languages spoken in the study districts (*Ateso, Lugbara* and *Madi*). All in-depth interviews were audio-recorded. To ensure quality, researchers discussed experiences, challenges and lessons learned in daily briefing and debriefing sessions while in the field with CR.

The document and record review was carried out by PM concurrently with the first round of qualitative data collection. At each selected facility, the following records for the periods May to October 2012 and May to October 2013 were reviewed:

- ANC register to establish number of first (IPT1) and second (IPT2) doses of IPTp provided and number of first (ANC1) and fourth (ANC4) visits;
- Monthly reports filed by the facility to the district level to compare IPTp and ANC data reported to the district level with data recorded in the facility’s register;
- SP stock cards to determine availability of SP for the provision of IPTp.

Review of records covering two equivalent 6-month periods was considered sufficient to allow the team to draw conclusions in terms of trends unaffected by seasonal variances. The researcher also noted gestational age of ANC attendees at the time of IPTp administration. However, this information was not systematically captured by the researcher. Gaps and inconsistencies in the reviewed documents were discussed informally with health workers while at the facility.

#### Data collection tools

Interviews were conducted using semi-structured interview guides (see Additional files 1, 2, 3, 4), which were developed by CR, KG, RK, and SGS based on a thorough review of the literature and experience implementing malaria in pregnancy prevention interventions. Interview guides were pre-tested, except for the guide for interviews with opinion leaders, which was largely based on guides used previously with the other target audiences. Table 3 summarizes the topics relating to supply-side challenges covered by the interview guides for the different target audiences. Note however, that all interview guides contained open-ended questions about respondents’ general perceptions of IPTp and the reasons behind the low uptake of the service, so respondents were not limited to elaborating on the topics covered by specific questions only.

**Table 3:** Supply-side topics covered by interview guides

Topic	District health officials	Health workers	Women	Opinion leaders
Role/responsibilities	X	X	–	X
Stakeholders and coordination	X	–	–	–
Policies and guidelines	X	X	–	–
Provision of ANC services	–	X	X	X
Supply chain	X	X	X	X
Capacity building	–	X	–	–
Health worker knowledge and perceptions of IPTp	–	X	–	–

X denotes that this topic was covered by a specific question or probe in the interview guide

For the document and record review, Excel spreadsheet software (Microsoft Corp) was used to capture information from facilities’ registers and reports.

#### Transcription and translation

Interviews conducted in English were subsequently transcribed verbatim, whereas those conducted in a local language were translated directly into English from the audio recording by the researcher who had conducted the interview. Field researchers were also required to take notes during the interview and to include non-verbal clues in the transcripts.

### Data analysis

Interview transcripts were managed using NVivo 10 qualitative analysis software (QSR International) and analysed by theme. An initial coding frame was developed by CR, KG and RK based on insights from a literature review and preliminary analysis of a sample of five interview transcripts with different types of respondents. CR subsequently coded all transcripts, discussing and agreeing proposed changes and additions to the original coding frame with KG and RK. Themes were analysed by code and summarized in a comprehensive study report. Data collected for the document and record review were synthesized and analysed by PM, and subsequently reviewed and discussed with CR, KG and SGS.

## Results and discussion

### Stakeholders and coordination

District officials in Eastern region listed a range of non-governmental stakeholders with an interest in ANC and malaria in pregnancy. They emphasized the positive role implementation partners play particularly with regard to providing resources, commodities, capacity building, and monitoring and evaluation. While planning and coordination mechanisms between districts and implementation partners were generally characterized as strong and well established, it was also repeatedly pointed out that there is no such coordination involving stakeholders from overlapping disease programmes such as HIV:

District official 1*: If*—*maybe*—*majority of them*—*like HIV. That’s where they now are. They are there. If maybe we could talk with them and integrate it there. Because malaria has an effect with HIV positive persons. If they talk to*— *maybe they could integrate. But most of their activity that I see, there’s no malaria.*

This echoes concerns raised in the literature that failure to create and maintain strong links between disease-specific programmes can lead to fragmented and ineffective service delivery [17]. In the case of IPTp, this is particularly significant as it cuts across the remit of malaria, maternal health and potentially other stakeholders, such as those focused on HIV or immunization [18].

District-level respondents in West Nile on the other hand generally reported a lack of support from implementation partners and pointed out that there is little coordination between the districts and the few implementation partners that do have a presence in the region:

District official 5*: Well* – *the organization which, err, handles aspect of* – *specific organization which addresses aspect of malaria in pregnancy is* – *not so far well streamlined.*

### Policies and guidelines

#### Consistency and relevance of guidelines

For policies to be implemented effectively, they need to be communicated and interpreted in a consistent manner across the health system [18]. A review of national-level malaria in pregnancy documents in Uganda carried out in 2013 revealed that guidance with regard to timing and dosage of IPTp was often inconsistent, with some documents specifying the timing of doses in terms of weeks of pregnancy, while others use months and some do not specify timings at all [12]. It was also not clear whether the time windows specified for the provision of IPTp are mandatory or whether they merely indicate when IPTp should ideally be administered. Similarly, there was a lack of clarity as to whether the guidelines define two as the maximum or simply the recommended number of doses a woman should receive.

None of the district officials and health workers interviewed was aware of the existence of conflicting guidelines. Most stated that the guidelines are clear and easily understood. Only one district official implied that ambiguous or outdated guidelines may cause confusion among health workers over when to offer IPTp to pregnant women attending ANC:

District official 2*: Then maybe one can receive that one who is at 36. What do I do? They told me 28. You are late. You have missed out.*

Even though the majority of district officials and health workers were not aware of the issue, the existence of inconsistent IPTp guidelines is likely to contribute to missed opportunities for the provision of the treatment to pregnant women, as health workers may not provide IPTp to women they incorrectly believe not to be eligible or because they prefer not to provide it because they are unsure about whether a woman is eligible. Similarly, using guidelines that are not in line with current WHO recommendations means that women will not receive optimum protection from malaria in pregnancy. It has been shown that inconsistent, complex or outdated guidelines contributed to low IPTp coverage in Tanzania and Mali [19, 20].

#### Male involvement in ANC

The Republic of Uganda’s Ministry of Health aims to encourage male partners to accompany pregnant women to ANC. Many respondents across all types of respondents suggested that this can lead pregnant women to assume they will not receive services if they cannot attend with their partners. It was also reported by respondents from all target audiences that some women will delay attendance because their partners are unable or unwilling to accompany them:

Opinion leader 5*: Sometimes when the pregnant women go without their partners, they are sent back and such a woman will refuse to go again unless the partner is there*—*and when the partners is far from her, the woman will not even step the health [facility] again.*

Several opinion leaders reported that women who did not attend with their husbands had been denied ANC services by health workers. While none of the women interviewed confirmed this, several indicated that they had been treated differently depending on whether or not their partners had accompanied them. For example, they reported that women attending with their partners had been seen to first, while those attending without their partners had been made to wait:

Woman 5: *The two visits I made with my husband we were attended quickly and we didn’t take long at the facility, but when I went alone, I took long, because those with partners are attended first.*

Male involvement in maternal and child health is a crucial factor in improving maternal and child health services [21] and should be encouraged, but health workers should be discouraged from giving preferential treatment to women who attend with their partners, which may not be possible for many.

### Provision of ANC services

#### Availability and accessibility of ANC services

Despite established standard procedures, ANC service delivery appeared to differ from facility to facility in the units visited. While some health facilities were said to provide daily ANC services in line with national guidelines, health workers, women and opinion leaders reported or implied that other facilities only offer ANC on certain days of the week, focusing on other services like family planning or immunization on the remaining days:

Woman 16: *They said each institution has different rules and regulations and at such a time they do other things, not to attend to mothers now.*

District officials stated that this was particularly common in lower-level facilities, some of which reportedly do not offer ANC services at all due to a lack of funding or qualified staff.

Many opinion leaders pointed out that low staff levels frequently lead to midwives being unable to attend to all ANC clients on a given day and refusing services to some women:

Opinion leader 2: *So these women from the village, they come footing up to the dispensary. Now, reaching there, they will get the nurses already selected twenty. The first twenty* –

Opinion leader 1*: Are the ones they handle.*

Opinion leader 2: *They will tell the majority, “Go back and come back next week.” […] Because now, for them they will always select the first twenty.*

Several opinion leaders related that midwives sometimes refuse to attend to women they perceive to be inadequately dressed:

Opinion leader 2: *You know like these village women, they come on their skirts, what* – *like that* [indicates tight fitting skirt]. *So these nurses want, “You come when you are putting on your maternity* [refers to a wide, loose-fitting skirt conventionally worn by pregnant women]. *You have a leso* [a cloth wrapped around the waist]*, we will attend to you.”*

It was suggested by both district officials and opinion leaders that some women may delay ANC attendance to avoid embarrassment or because they do not have the resources to buy a maternity dress, which may result in late or irregular ANC attendance and hence missed opportunities for the provision of IPTp. Non-availability or refusal of ANC services by health workers not only leads to women not receiving vital services, including IPTp, when they visit the health facility, but those practices are also likely to have a negative effect on uptake of ANC and IPTp in the longer term, as they will shape women’s and communities’ perception of ANC services.

#### Job satisfaction

Opinion leaders, women and district officials speculated that low job satisfaction among front-line health workers may contribute towards poor attitude and unprofessional behaviour among ANC staff. While district officials suggested that staffing levels were generally adequate, many health workers stated that there were too few staff in ANC and that midwives’ workload was immense. This is in line with reports of widespread dissatisfaction with working conditions among health workers in the literature [22, 23].

Many respondents from all target audiences stated that they had heard of cases of unprofessional behaviour on the part of health workers. They frequently commented on the effect of poor staff attitude on women’s perceptions of ANC, linking negative experiences to low uptake or late ANC attendance:

District official 1: *Maybe also the other factor is*—*I cannot fear to mention this*—*is the attitude of the health workers*—*the ANC midwives. Their treatment to the* – *to the mothers. Also*—*possibly also can make them shy away.*

Disrespectful and rude health worker behaviour was reported specifically in the case of women who miss ANC appointments, as well as for women who are perceived as initiating ANC too early or too late. Since those are particularly likely to miss out on the recommended number of doses of IPTp, health workers’ brusqueness with this type of client is worth noting. This reflects findings of previous studies conducted in Uganda [24], which cited instances of mistreatment of clients by health workers and found that clients’ satisfaction levels with health service are low. However, most of the women interviewed, regardless of whether or not they had been selected with the help of a health worker, stated that they had generally been treated politely and respectfully during their own ANC visits:

Woman 2: *In this health centre, you are welcomed and treated well. They mind of your health and the wellbeing of pregnant women.*

#### Incentives

Many respondents referred to the provision of free mosquito nets, usually during a pregnant woman’s first visit, as a major incentive to attend ANC:

District official 5: *But, you’ll realize that some of these indicators just improve*—*sometimes they just improve by themselves when a cer*—*especially when certain incentives are there. Like, recently, there is net*—*because of the net, the enrolment has started changing.*

This was confirmed by many of the women interviewed:

Researcher: *Is there anything else that encouraged you to go for ANC that first time?*

Woman 3: *Yes, I went to the hospital they gave me my net I came back to sleep under my net.*

A number of respondents from one study district reported that mosquito nets for ANC had not been available for a number of months. The shortage was repeatedly linked to a universal net distribution campaign, which may have resulted in available nets being channelled away from ANC. Respondents expressed concern that this campaign could result in lower ANC uptake as receiving a net will no longer be seen as an incentive for attending ANC. Other respondents felt that the lack of nets for ANC could lead to reduced protection from malaria for some pregnant women, as the nets distributed through the campaign might be reserved for use by men in a context where pregnant women traditionally do not share a bed with their partners. Continuing the provision of incentives was one of the most frequently mentioned recommendations in terms of maintaining high levels of ANC attendance.

### Supply chain

#### Availability of SP

District health officials and health workers in both study regions reported that the supply chain had been significantly strengthened through the efforts of the Ministry of Health and implementation partners in recent years and, as a result, stock-outs of SP no longer occur. Women and opinion leaders also reported that drugs for IPTp, unlike many drugs for other purposes, were typically available in the health facilities. This was confirmed by the review of SP stock cards, which did not find stock-outs during the 6-month period under review in the public facilities visited. This is a very encouraging finding since shortages and stock-outs of SP had been identified as major bottlenecks to IPTp provision in many countries, including Uganda [11, 22, 25]. The findings from this research are in line with those of a recent comparative study in Ghana, Kenya and Malawi, which did not find evidence of drug shortages [26].

However, PNFP facilities do not seem to have benefited from supply chain strengthening efforts in equal measure. Under the current system, National Medical Stores, an autonomous government corporation tasked with ensuring drug supply in the public health sector, provides SP free of charge to public facilities, but private and PNFP facilities are required to procure SP from Joint Medical Stores, a not-for-profit wholesale enterprise that sells pharmaceuticals and equipment to the private health care sector. As funds to procure SP are often limited, frequent stock-outs of the drug were reported by health workers at the PNFP facility visited for this study. This was also confirmed more generally by district health officials for PNFP facilities in their district. Where SP is available, health workers at this facility described how they are forced to choose between two scenarios: either they require women to pay a fee for the provision of IPTp (against a national guideline which requires facilities to provide IPTp free of charge) or they provide IPTp for free to clients, but at a financial loss to the facility. Either scenario is likely to negatively affect IPTp uptake, particularly given the significant role of the private health care sector in Uganda [27].

A number of respondents emphasized the negative impact of non-availability of drugs on women’s and communities’ perceptions of ANC. In Eastern region, the term used for attending ANC in the local language literally translates as *going to take the medicine*, so in this context failure to provide medication may be equated with failure to provide good ANC in general:

Health worker 7: *Because now our mothers are used* – *[…] It motivates them that when you are going to a facility, there is that saying* – *they say that, “alosingo amat ekiya”* [Ateso for ‘I am going to take medicine’], *so if she goes away without any medicine, it will imply that her visit was not fruitful, so it demoralizes them.*

#### Availability of water and cups

District officials and health workers overwhelmingly reported that water, jerry cans, cups, and water purification tablets had been provided in sufficient quantities. The majority of the women interviewed in both study regions also reported that clean cups and water had been available when they were offered IPTp. This is another encouraging finding, as research carried out in Uganda in 2011 had found that, while 74 % of facilities had safe water ready for use in ANC, only 47 % had adequate cups [28]. Lack of safe water and cups has also been reported in studies in other African countries [22, 29].

### Capacity building

#### Individual supervision

Formal supervision or mentoring mechanisms for ANC staff were only reported by health workers at one of the eight facilities visited. More typically, health workers related that they can discuss complex cases with senior staff on an *ad hoc* basis if required. However, several health workers felt that such opportunities were rare:

Health worker 11: *Actually I don’t know how I should say it has not really been so much there. It is just once in a while.*

#### Training

In terms of receiving formal classroom training on IPTp, health workers were evenly split: about one-third indicated they had received training on IPTp; one-third said they had not been trained on IPTp specifically, but IPTp had been covered as part of a broader training, for example on treatment of malaria; and, one-third stated they had never received any formal training on IPTp. There was a consensus among health workers and district officials that training is an important factor in ensuring quality of service delivery, as it enables health workers to refresh their knowledge and keep track of scientific progress. However, it was also pointed out that classroom training can be disruptive to service provision. A number of health workers expressed a preference for less disruptive training methods such as on-the-job training or mentoring:

Health worker 11: *I think the training would be better on job here* – *not carrying people somewhere, but it would be better here just on job. Like it is an antenatal day, mothers are also there* – *we get mothers so* – *kind of mentorship I think it would be better*.

Lack of supervision and training on IPTp is likely to contribute towards health workers’ poor understanding and knowledge of current IPTp guidelines. This confirms the results of several studies that identified a lack of effective training and individual supervision as barriers to the provision of IPTp [22, 30]. Conversely, training health workers has been shown to increase uptake of IPTp [31]. In Uganda, a review of relevant materials found that no pre-service education materials for midwives covering malaria in pregnancy or IPTp existed in 2013 [12].

### Health worker knowledge and perceptions of IPTp

#### Health worker knowledge

When asked to outline the current guidelines for IPTp provision, it transpired that there was wide variation between health workers, particularly in terms of their knowledge and interpretation of the recommended timing of IPTp administration. Most health workers correctly specified that the first dose of IPTp should not be given in the first trimester, with 16 weeks mentioned by a majority of respondents as the lower limit. Week 20, 24, 26 and 28 were variously mentioned as the upper limit. There was similar variance in terms of the timing of IPT2, where the lower limits mentioned ranged from week 16 to week 34, with upper limits mentioned ranging from week 28 to 36. Very few health workers pointed out that IPTp can be given up to the time of delivery and two explicitly stated that IPTp should not be given during the later stages of a woman’s pregnancy.

Most health workers implied that they treat the time windows specified by the guidelines as recommendations only. However, several health workers suggested that the time windows are mandatory, which means opportunities for the provision of IPTp may be missed if women present for ANC at other times. Similarly, the majority of health workers appeared to interpret the current guidelines as specifying that a woman should not receive more than two doses. In light of current evidence that three or more doses of SP are associated with fewer low birth weight deliveries compared to the standard two-dose regimen [32], this means women do not currently receive optimum protection from the effects of malaria in pregnancy. Health workers’ confusion about timing and dosing has been cited as a barrier to IPTp provision by a number of studies in a range of African countries [33, 34]. Inconsistent and unclear policies and guidance [12] are likely to contribute to the inadequate knowledge and differing interpretations of the guidelines among health workers.

Observations made as part of the document and record review revealed that provision of IPTp appears to vary between facilities as well as between individual health workers. In some health facilities, pregnant women were generally not offered IPTp until after week 22 of their pregnancies even if they had initiated ANC earlier. Similar variations were found at the later stages of pregnancy, with women not being offered IPTp if they reported or returned after week 36. All the practices observed create missed opportunities for the provision of IPTp, which is considered safe from week 13 up to the time of delivery, despite previous concerns about the safety of administration of SP late in pregnancy [5].

#### Safety and efficacy

All district officials and health workers demonstrated an understanding of malaria in pregnancy as a health risk to mother and child. They generally agreed that in order to maximize protection, pregnant women should take preventive medication in addition to practicing protective behaviour, such as sleeping under a mosquito net. While most district officials and health workers were broadly supportive of the concept of IPTp, some appeared to have reservations with regard to its efficacy and safety. This was based mainly on the observation that cases of malaria in pregnancy persist despite IPTp administration over several years:

Health worker 11: *But about how efficient, I have some questions about it and am not really very sure if it is really efficient. As I told you before, much as mothers take it, they still suffer from malaria. So my concern is in that area how efficient it works.*

A small number of health workers also expressed concerns about the side effects of SP, particularly if taken on an empty stomach. Several health workers mentioned that SP can also be used for the treatment of symptomatic malaria; however, a few pointed out that it is no longer used as a first-line anti-malarial. One interviewee indicated that the reasons behind discontinuing SP for the treatment of malaria while retaining it for IPTp were not clearly communicated, raising doubts with regard to the drug’s safety and efficacy. Two health workers in West Nile on the other hand indicated that they still use SP to treat symptomatic malaria because they feel it is more efficacious or better tolerated than other available anti-malarial drugs.

About half the health workers interviewed stated that they were not aware of or had never been confronted with safety issues or side effects related to IPTp administration. The other half indicated that women did occasionally experience side effects after receiving IPTp and there were also concerns over allergic reactions to SP. However, most of the health workers who voiced concern elaborated that side effects and allergic reactions were rare and typically mild:

Health worker 12: *Well, there is this slight side effect of having maybe headache, dizziness*—*the dizziness is there. Maybe nausea*—*feeling like vomiting. But we have not got very serious side effects which is adverse effect*—*adverse* —*which is worse side effect. We have not got it. But this one of nausea*—*feeling like vomiting*—*abdominal pain*—*they complain. But it’s mild. It’s mild. Not very serious yet.*

Only two midwives stated that the side effects can be serious, one of them because she herself had experienced them after taking SP.

In order to ensure health workers’ compliance with IPTp provision guidelines, it will be important to provide information about the efficacy and safety of SP. While none of the health workers interviewed stated that they do not provide IPTp because they doubt the drug’s efficacy and safety, it cannot be ruled out that this may contribute to low IPTp coverage.

#### Offering IPTp

All health workers interviewed reported that if SP is available and they have determined a client’s eligibility, they always offer IPTp. A majority of the women interviewed also indicated that they had been offered IPTp when they attended ANC. However, the three women who were interviewed because they had not completed a full course of IPTp related that they had not been offered the treatment by health workers on at least one occasion, despite regular ANC attendance. With regard to supply-side issues, this was the only noticeable difference between the responses given by the women interviewed during the two rounds of data collection. Health workers did not explain to those women who did not receive IPTp why the service had not been offered.

Researcher: *What reasons were you told for not getting the tablets?*

Woman 19: *The nurse did not tell me why I didn’t receive the tablets.*

Researcher: *There was nothing you were told completely?*

Woman 19: *The nurse only examined me and there after she did not tell me the reason for not getting the tablets.*

Note however, that self-reported administration of IPTp may be unreliable. A recent study in Uganda found that only about one-third of women who self-reported having used IPTp tested positive for SP in the blood stream [35].

Health workers and district officials described a number of scenarios where IPTp might not be provided. For example, it was suggested by one respondent that health workers might prioritize provision of IPT1 over provision of IPT2 when stock levels of SP are low. A district official speculated that some health workers might not provide IPTp if women initiate ANC late during their pregnancies—either to punish them for late attendance or because they think SP is not safe to be administered during the final weeks of pregnancy. Many health workers stated that they do not offer IPTp to women who report having ‘reacted’ to IPTp during previous pregnancies. However, from respondents’ examples, it appears they are referring to mild side effects rather than potentially life threatening allergic reactions:

Health worker 8: *We got one who said in the previous pregnancy she took it and it really worsened her condition* – *as in she started vomiting* – *that thing* – *and you know, so we just* – *what we did was to give her the information* – *ok to give her the benefits of taking IPTp, but we didn’t force her to take.*

In the context of poor knowledge of the IPTp guidelines and lack of adequate training, this might reflect a lack of knowledge of common side effects and contra-indications of IPTp, a challenge cited in a recent study in Ghana [36]. In order to maximize provision of IPTp, it will be important to educate health workers about differentiating between mild and serious side effects. This is particularly relevant as health workers frequently reported that women who are reluctant to take IPTp out of fear of side effects can be persuaded to accept the treatment if health workers provide information about the possible side effects and assure women that they are typically mild:

Health worker 9: *Of course during health education talks they can ask you questions. In case when somebody develops side effects like rush and they*—*and in case somebody develop*—*feel sometimes nausea, but with the help of health information given to them, they accept.*

#### Observing DOT

The majority of health workers and opinion leaders reported that IPTp is routinely provided under the supervision of midwives as DOT. However, several health workers reported that they sometimes do not observe DOT, particularly if their workload is high or if women request to take the tablets home because they have not eaten and do not want to take the medication on an empty stomach:

Health worker 12: *Well, practically what we do is when these people come for antenatal, we tell them to take it at DOT. […] But the challenge is some mothers*—*they have come minus having breakfast. So they fear taking the drugs minus food. We tell them, “No you can take and go home, then have food.” But some insists they are going to take it, they first want to eat then they take the drugs, which is also right.*

This practice appears to be particularly widespread in West Nile. A district official and two facility in-charges in this region confirmed that they were aware that midwives sometimes ask women to take the tablets at home, particularly if they are busy. This practice is problematic as compliance with DOT has been shown to increase IPTp uptake [37]. Midwives should be educated about the benefits of DOT and should be reminded to strictly observe the guidelines.

### Monitoring and evaluation

#### Data recording and reporting practices

Effective and consistent tracking and reporting of interventions helps to ensure policy makers, implementers, donors, and key stakeholders are kept informed of the progress towards achieving desired objectives, indicating the impact of current activities and identifying areas that require improvement [38]. While data relating to ANC and IPTp are routinely recorded in all facilities visited, the document and record review found that recording practices differed widely between facilities and individuals. For example, non-provision of IPTp is recorded using a variety of symbols and abbreviations, being variously denoted by a dash <->, a tick <✓>, <NT>, <ND>, or <N/A> in the IPTp column of the ANC register. Similarly, while <NA> stands for ‘not available’ in some cases, in others it is used to denote ‘not applicable’ or ‘IPTp completed’ (i.e., client previously received two doses of IPTp). Moreover, while a tick stands for ‘IPTp given at this visit’ in some facilities, in others it signifies ‘IPTp was given in the past’. Many health workers also reported that standard HMIS recording tools, such as maternal passports and ANC register books, are often out of stock, requiring staff to use poor photocopies or manually drawn registers instead, which may be inconsistent with the standard tools.

Several midwives indicated that their recording and reporting duties significantly add to their workload. However, most midwives interviewed commented that they view data recording and reporting as routine work, which does not pose any significant challenges:

Health worker 5: *Yes*—*and also with our reporting. […] So it’s a daily thing*—*so it is something which is every day. So I think you cannot even forget it since you’ve started it and something which is in record that you are supposed to do it on daily basis whenever a mother*—*it’s a routine.*

At the district level, aggregating facility-level data and reporting to the national level is the responsibility of an HMIS focal person or biostatistician. Facility-level data are entered into the District Health Information System (DHIS 2) online reporting tool, which connects district health offices with the Ministry of Health Resource Centre. The DHIS 2 data entry form automatically rejects certain entries as erroneous. For example, it does not allow the figure for provision of IPT1 to be greater than the one reported for ANC1 and the figure for provision of IPT2 must not be greater than the one reported for provision of IPT1 in any given month. However, all the scenarios rejected by the system are logically possible and do in fact occur quite frequently. Discussions with data managers at district level revealed that they resort to adjusting the reported figures to match the data entry system’s requirements when faced with this challenge, thereby falsifying the reports received from the facilities:

District official 7: *Okay now, secondly, when we get it*—*like we*—*we do*—*we have ANC1*—*ANC first visit is 218. IPT1 is rated maybe 300. That one can’t happen. So we reduce that one to this* [indicates act of replacing one figure for another].

#### Data accuracy

Both district officials and facility health workers overwhelmingly stated that they believe facility and district-level data to be fairly accurate. There was, however, a clear mismatch between health workers’ perceptions and the results of the document and record review, which found that both under and over-reporting with regard to all indicators reviewed occurred in all facilities visited. In some cases, figures reported to the district varied from data recorded in the facility’s register by more than 500 %. For example, in July 2013, a health centre II recorded three ANC4 visits, but reported 25 (variance of 733 %). Similarly, in the same month, a hospital recorded five women who received IPT2, but reported 34 (variance of 580 %). While both under and over-reporting on a smaller scale of up to 25 % occurred very frequently, high variances in excess of 50 % tended to be cases where a much higher number was reported compared with what had been recorded in the register, maybe because health workers feel under pressure to report high uptake figures in an attempt to present their work in a positive light, as suggested by one district official:

District official 1: *Then at the end of the month, you say, “Ah, what do I do?” That’s where I’m*—*the other*—*maybe the other issue of exaggerating data comes in. You can feel, “Ah, now, I need to be performing better. Well, my mothers have been*—*and the numbers have been ok. Then this time, what will they think, hm?” I think that’s what I*—*my mind takes me that maybe that’s where it sometimes*—*you get these figures interchanged.*

**Fig. 3:**
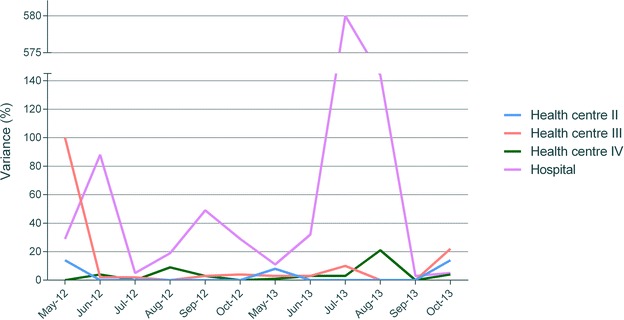
Reporting variance for the provision of IPT2 in four health facilities, May–October 2012 and May–October 2013

Discrepancies between recorded and reported data tended to be greater at higher level than at lower level facilities, probably because of the larger data volumes handled by higher level units (see Fig. 3). In comparable facilities, discrepancies were found to be lower in Eastern than in West Nile, possibly as a result of the higher levels of implementation partners’ support in Eastern region. The poor accuracy and quality of data recording found through the document and record review reflect the conclusion of WHO’s assessment of health facility data in Uganda [15].

Where health workers acknowledged that, on occasion, data inaccuracies do occur, they typically ascribed them to high workload:

Health worker 3: *Challenges when women are very many, you get tired and sometimes, recording, you need to record, you need to go for other things, examinations.*

Poor data management skills and lack of training were also mentioned. Some respondents pointed out that non-compliance with DOT might obscure actual IPTp uptake figures, as women might not actually take SP, while health workers are likely to record provision of IPTp in the register. Many health workers noted that data accuracy did not appear to be seen as a priority, as the criteria used for assessing facilities’ monthly data reports at the district level are promptness and completeness. Districts do not commonly provide feedback to the facilities with regard to the accuracy of recorded data submitted.

The results presented above suggest that coverage rates recorded and reported should be treated with caution, as there appear to be sources of error and inaccuracy along the recording and reporting chain. In Uganda, poor data quality and reporting practices have been identified as important challenges to effective monitoring and evaluation of malaria interventions [11] and HMIS has been singled out as a priority area requiring improvement [39]. Furthermore, it should be noted that there is currently no system for collecting and integrating data from the private sector into HMIS [11].

## Conclusions

This research confirmed that a range of supply-side issues continue to impede uptake of IPTp in Uganda. While most barriers to IPTp uptake discussed by the different types of respondents applied equally in both study regions, where differences between the regions were evident, for example, with regard to reserving SP for IPTp and observing DOT, supply-side issues appeared more pronounced in West Nile than in Eastern region. This probably reflects the lower levels of implementation partner support this region has received.

There was a high level of agreement on supply-side barriers among all target audiences. District health officials tended to emphasize issues relating to stakeholder coordination, provision of ANC and the supply chain, whereas health workers tended to highlight a lack of training opportunities. Women and opinion leaders on the other hand stressed the negative impact of health workers refusing services to women attending ANC. Most challenges were mentioned to a greater or lesser extent by all target audiences however, and the design of the question guides for the different target audiences anticipated priority challenges for each type of respondent, which may have biased responses with regard to the relative importance attached to specific issues. It was also noticeable that there was generally a lack of awareness of conflicting IPTp guidelines by district level officials and health workers and an inadequate knowledge of guidelines by health workers.

The document and record review revealed that, contrary to district officials’ and health workers’ perceptions, data recording and reporting may be poor, with sources of errors along the recording and reporting chain. This limits policy makers’ and service providers’ ability to assess the scope of the problem and react appropriately to trends and challenges.

Based on the results of this study, the following actions are recommended to overcome key supply-side barriers:

- *Stakeholders and coordination* Governments and implementation partners should increase efforts to build strong linkages and improve integration in order to ensure that health programmes reach those most in need.
- *Policies and guidelines* In its latest policy recommendation, WHO acknowledges that “confusion among health workers” contributed to a slowing down of efforts to scale up IPTp and expresses the hope that the new simplified guidelines will help increase uptake of IPTp through avoiding ambiguity in terms of number of doses required and ideal timing [16]. While Uganda adopted this recommendation in May 2016, ensuring that health workers are aware of the policy change and implement it in their day-to-day job should be treated as a matter of priority. In the light of reports of preferential treatment for women attending with their partners, the national-level policy, which encourages male ANC attendance, should be clarified, emphasizing the imperative to provide ANC services to all women equally, regardless of whether or not they attend with their partner.
- *Provision of ANC services* ANC should be offered consistently at all suitable health facilities in line with national policy. Governments must provide for adequate staffing levels, but health workers also need to observe professional and respectful behaviour towards all ANC clients and ensure all pregnant women attending ANC have equal access to services. In particular, this applies to women who miss appointments, present late for ANC or are perceived to be inadequately dressed. Early and regular ANC attendance should be encouraged through the provision of incentives, such as mosquito nets.
- *Supply chain* While only one PNFP facility was visited and this type of service provider was therefore under-represented in the sample, the issue of persisting stock-outs of SP in PNFP facilities was confirmed more widely by district health officials. In the interest of equity, the Government should enable PNFP facilities to offer IPTp to pregnant women by providing SP free-of-charge. In addition to ensuring IPTp is provided to women who attend ANC, ensuring availability of SP will also positively influence communities’ attitudes towards ANC and hence help to maintain high ANC uptake.
- *Capacity building and health worker knowledge and perceptions of IPTp* To improve health worker knowledge of IPTp guidelines, opportunities for training and supervision should be intensified. Training should emphasize that it is safe to take SP on an empty stomach and that women should be encouraged to take IPTp as DOT, even if they ask to take the drug home, for example, because they have not eaten. Clear guidance with regard to distinguishing between mild and severe side effects should be provided, emphasizing that women should still be encouraged to take SP if they have previously experienced mild side effects. Taking into account health workers’ preference for non-disruptive training, innovative methods such as peer learning, mentoring or the use of mobile technology should be trialled in addition to traditional classroom training.
- *Monitoring and evaluation* In order to improve the quality and reliability of data relating to ANC and IPTp at the facility and district level, supply of standard recording and reporting tools to all health facilities needs to be ensured. It will also be necessary to improve health workers’ data management skills, provide clear guidelines with regard to recording practices and ensure data entry forms are designed to capture all plausible scenarios. In addition to assessing completeness and timeliness of reported facility-level data, districts should also provide feedback with regard to data accuracy.

## Additional files

10.1186/s12936-016-1405-4 Interview guide district health officials.
10.1186/s12936-016-1405-4 Interview guide health workers.
10.1186/s12936-016-1405-4 Interview guide women (English).
10.1186/s12936-016-1405-4 Interview guide opinion leaders.

## Abbreviations

COREQ: consolidated criteria for reporting qualitative research
ANC: antenatal care
ANC1: first antenatal care visit
ANC4: fourth antenatal care visit
DHIS 2: District Health Information System
DOT: directly observed therapy
HC: health centre
HIV: human immunodeficiency virus
HMIS: Health Management Information System
IPT1: first dose of IPTp
IPT2: second dose of IPTp
IPTp: intermittent preventive treatment in pregnancy
PNFP: private not-for-profit
SP: sulfadoxine–pyrimethamine
WHO: World Health Organization
