# Supplementary material for: Assessing supply-side barriers to uptake of intermittent preventive treatment for malaria in pregnancy: a qualitative study and document and record review in two regions of Uganda
Source: Malar J. 2016 Jul 4;15:341. doi: 10.1186/s12936-016-1405-4 (PMC4932731; doi:10.1186/s12936-016-1405-4)
Supplement: Supplementary file 1 — 10.1186/s12936-016-1405-4 Interview guide district health officials. [file 12936_2016_1405_MOESM1_ESM.pdf]

Interview Guide – District Health Officials

**Interview Code**	
**Interviewee Code**	

**Name of Interviewer**	
**Date of Interview**	
**Time Started**	
**Time Finished**	
**Location**	
**Job Title**	
**Time in Current Position**	

Introduction:

Good morning/ good afternoon

My name is [name]. I work as a researcher for Malaria Consortium, a not-for-profit organisation that specialises in the prevention, control and treatment of malaria and other diseases. We are currently carrying out research that will help us to find out how we can tailor our programmes so more people benefit from what we do.

We have put together an information sheet that tells you more about the research we do, why you've been selected as a suitable interview partner and what happens if you agree to take part in the interview.

[give time to read information sheet]

Do you have any questions about this information? As the information sheet explains, you do not have to take part in the interview if you prefer not to.

If you agree to be interviewed, I will audio record the conversation and take notes throughout the interview. This will help me remember what you are telling me. Everything you say will be strictly confidential. Your replies will only be shared with the other researchers working on this study and will be identified by a number, not your name. Can I please ask you to also read this consent form?

[give time to read consent form]

Do you have any questions about the information in this form? If you consent to being interviewed, can I please ask you to sign the form?

[interviewee and researcher sign consent form]

Thank you very much for agreeing to take part in this interview. I will now switch on the audio recorder. Remember that you do not have to answer questions you don't feel comfortable with and can end the interview at any time.

Let me begin by asking you a few questions about your job.

- What's your name?
- What is your job title?
- How long have you been in your current role?

1. Can you please summarise your role and responsibilities?

1.1 What are your role and responsibilities with regard to malaria in pregnancy?

1.2 Which malaria in pregnancy interventions are being implemented in your district?

1.3 How important is malaria in pregnancy in your role compared with other areas you are responsible for?

1.4 How important is the provision of IPTp compared with other areas you are responsible for?

2. Can you please summarise the strategies, policies and guidelines with regard to malaria in pregnancy and IPTp which are currently in place in Uganda?

2.1 In which documents can the strategies, policies and guidelines be found?

2.2 Have the policies and guidelines changed recently?

3. According to the 2011 *Uganda Demographic and Health Survey*, the share of pregnant women who had received at least two doses of Sulphadoxine-pyrimethamine (SP or Fansidar), at least one of which during an antenatal care visit, was 24.5%. This compares with the President's Malaria Initiative target of 85% of pregnant women receiving at least two doses of IPTp by the end of 2015 and the Roll Back Malaria target of universal access and utilisation of malaria prevention measures, including IPTp, by 2015.

What do you think are the main reasons why targets with regard to IPTp uptake in Uganda are not being met?

3.1 What do you think are the main reasons why targets with regard to providing two or more doses of IPTp are not being met?

3.2 Does your district have a plan with regard to increasing IPTp uptake?

3.3 In your opinion, how could the national guidelines be changed to better address the challenges you identified?

4. Can you describe how policies and policy changes are communicated to the district level?

4.1 How well does this process work?

4.2 How could communication between the national and district level be improved?

4.3 Can you describe the systems and mechanisms that exist to ensure national policies and guidelines are implemented at the district level?

4.4 Are there any challenges in terms of implementing policy changes?

5. In terms of capacity and resources, which factors impact your ability to implement effective malaria in pregnancy and IPTp strategies, policies and guidelines?

5.1 Does the availability of financial resources affect the level of priority you are able to assign to malaria in pregnancy and IPTp provision compared to other areas you are responsible for?

5.2 Do recruitment policies and staffing levels affect IPTp provision?

5.3 Do you feel you receive sufficient support to enable you to efficiently implement IPTp provision in your district?

6. Who are the main stakeholders with an interest in IPTp provision in your district?

6.1 What are their respective roles and responsibilities?

6.2 Do you feel the roles and responsibilities of the various district-level stakeholders are well defined and understood?

7. How would you describe the collaboration between the various stakeholders in your district?

7.1 How established and effective are cross-stakeholder planning mechanisms?

7.2 Would you say some stakeholders are more involved in cross-stakeholder planning efforts than others?

7.3 What do you see as the main challenges with regard to coordinating and integrating activities of multiple stakeholders?

7.4 In your opinion, what could be done to improve coordination and integration between stakeholders?

8. Districts are at the centre of data recording and reporting, as they receive information from the facility level and report data to the national level.

Can you please describe the exchange of data between the facilities and the district level with regard to IPTp coverage and uptake?

8.1 In your opinion, is the data you receive from the facilities of good quality?

8.2 What do you see as the main challenges in terms of data accuracy and quality?

8.3 Are there any difficulties in terms of distinguishing between first, second and subsequent doses when reporting on IPTp provision?

8.4 How do you deal with cases where it is not clear which dose the reports are referring to?

8.5 In your view, how could the exchange of information between the facilities and the district be improved?

9. Can you please describe the exchange of information between the national and the district level with regard to IPTp coverage and uptake?

9.1 How well does it function?

9.2 Does the national level provide feedback on the data you provide?

9.3 Does the national level provide information on how your district is performing compared with other districts?

9.4 Would you find this information useful?

9.5 What could be done at the district level to strengthen the richness and quality of data you report to the national level?

10. The *Uganda Malaria Programme Review Report* notes that stock-outs of SP in antenatal care services have hindered the implementation of IPTp.

Do you feel it is possible to accurately predict and project required stock of SP at the district level?

10.1 In your opinion, what are the challenges around predicting and projecting required stock of SP?

10.2 Do you feel the facilities provide the district with sufficient information with regard to stock requirements, storage and tracking?

10.3 How do you become aware of shortages of SP at the facilities in your district?

10.4 At what points in the reporting and supply chain are delays likely to occur?

10.5 In your opinion, how could supply chain management be strengthened at the district level?

11. Sometimes, IPTp cannot be provided because there is no clean water or clean drinking cups are not available at the facility.

What can be done at the district level to ensure availability of commodities such as clean water and cups for IPTp under directly observed treatment (DOT)?

12. Other than the challenges we have discussed, can you think of any additional issues that need to be addressed in order to increase uptake and coverage of IPTp in your district?

13. Of all the challenges and barriers to effective prevention and control of malaria in pregnancies we discussed today, which do you think are the most relevant in terms of increasing uptake and coverage of IPTp?

14. What are your main recommendations with regard to increasing uptake and coverage of IPTp?
