# Supplementary material for: Assessing supply-side barriers to uptake of intermittent preventive treatment for malaria in pregnancy: a qualitative study and document and record review in two regions of Uganda
Source: Malar J. 2016 Jul 4;15:341. doi: 10.1186/s12936-016-1405-4 (PMC4932731; doi:10.1186/s12936-016-1405-4)
Supplement: Supplementary file 2 — 10.1186/s12936-016-1405-4 Interview guide health workers. [file 12936_2016_1405_MOESM2_ESM.pdf]

Interview Guide – Health Workers

**Interview Code**	
**Interviewee Code**	

**Name of Interviewer**	
**Date of Interview**	
**Time Started**	
**Time Finished**	
**Location**	
**Age of Interviewee**	
**Interviewee Job Title**	
**Time in Current Post**	

Introduction:

Good morning/ good afternoon

My name is [name]. I work as a researcher for Malaria Consortium, a not-for-profit organisation that specialises in the prevention, control and treatment of malaria and other diseases. We are currently carrying out research that will help us to find out how we can tailor our programmes so more people benefit from what we do. It was suggested by the in-charge at your facility that you might be a good person for us to speak to.

We have put together an information sheet that tells you more about the research we do, why you've been selected as a suitable interview partner and what happens if you agree to take part in the interview. Would you like to read it or would you prefer me or someone else to read it out for you?

[give time to read or read out information sheet]

Do you have any questions about this information? As the information sheet explains, you do not have to take part in the interview if you prefer not to.

If you agree to be interviewed, I will audio record the conversation and take notes throughout the interview. This will help me remember what you are telling me. Everything you say will be strictly confidential. Your replies will only be shared with the other researchers working on this study and will be identified by a number, not your name.

Can I please ask you to also read this consent form? I or someone else can read it out for you if you prefer.

[give time to read or read out consent form]

Do you have any questions about the information in this form? If you consent to being interviewed, can I please ask you to sign the form?

[interviewee and researcher sign consent form]

Thank you very much for agreeing to take part in this interview. I will now switch on the audio recorder. Remember that you do not have to answer questions you don't feel comfortable with and can end the interview at any time.

Let me begin by asking you a few questions about your job.

- What's your name?
- How old are you?
- What is your job title?
- How long have you been in your current role?

1. **Can you please describe the services you provide as part of antenatal care?**

2. **In your experience, how often do most women attend antenatal care during pregnancy?**

    2.1 Why do you think many women do not follow the recommended schedule of four antenatal care visits?

3. **Let's talk about one particular service you provide: protecting women from malaria in pregnancy.**

    Can you please summarise your role and responsibilities with regard to malaria in pregnancy?

    3.1 What do you tell women about malaria in pregnancy?

    3.2 What do you tell women about how they can prevent malaria in pregnancy?

    3.3 Do you offer health education talks at your facility?

    3.4 How are they organised?

    3.5 Do you talk about malaria in pregnancy in the health education talks you offer?

4. **I would like to know more about one specific malaria in pregnancy service: intermittent preventive treatment in pregnancy or IPTp.**

    Have you been given any training on IPTp?

    4.1 If yes, how long did it last?

    4.2 When did it happen?

    4.3 How useful and relevant to your job was the training you received?

    4.4 In your opinion, how could the training you been improved?

    4.5 Do you feel you need more training on IPTp?

5. **Are there opportunities for you to discuss difficult cases with regard to malaria in pregnancy and IPTp with a supervisor?**

5.1 If yes, how often do you have this opportunity?

5.2 How could your employer ensure you are provided with sufficient opportunities to ask questions relating to malaria in pregnancy and IPTp?

6. **Do you have access to job aids and guidelines that help you decide when or how often you should offer IPTp to pregnant women who attend your antenatal care clinic?**

6.1 How practical and easy-to-use are the job aids and guidelines you have access to?

6.2 In your view, how could the job aids and guidelines be improved?

7. **Can you tell me what your current guidelines say with regard to providing IPTp as part of antenatal care?**

7.1 Who is eligible?

7.2 When should it be offered?

7.3 How often?

7.4 Are there any scenarios when IPTp shouldn't be offered?

7.5 Would you say the guidelines are clear or have you been given conflicting guidance?

7.6 Are you ever unsure whether or not IPTp should be offered to a woman who's attending antenatal care?

7.7 If yes, what are the challenges in determining whether or not she is eligible?

7.8 Do you normally ask women to take the tablets under your supervision or do they take the tablets at home?

8. Have **the guidelines with regard to malaria in pregnancy and IPTp changed over the last three years?**

8.1 If yes, what has changed?

8.2 Was it difficult to change the practice of IPTp provision at your facility in line with the new guidelines?

8.3 How were the changes communicated to you?

8.4 In your opinion, how well were the changes to guidelines communicated to you?

9. **How do you feel about giving pregnant women drugs to prevent them from getting malaria?**

9.1 If a pregnant woman sleeps under a net, do you think there is still a need for her to take medication to prevent malaria?

9.2 Do you normally offer IPTp to eligible women who attend antenatal care?

9.3 If not, why not?

9.4 In your experience, do women tend to accept IPTp when offered?

9.5 When women refuse to take the tablets, what are the main reasons women give?

10. Do you keep a record of how many doses of IPTp you have administered?

10.1 Can you tell me how and where this information is recorded?

10.2 Do you ever find filling in the antenatal care register is difficult or challenging?

10.3 How do you find out how many doses of IPTp a woman has previously received, for example if women attend different antenatal care providers or they forget to bring their antenatal care cards?

10.4 How do you think data recording could be improved at your facility?

11. According to a report published by the Ugandan government, facilities occasionally run out of the drug used for IPTp. This means some women cannot be offered IPTp when they attend antenatal care on days when the drug is not available.

Has this ever happened at your facility?

11.1 If yes, how often does this happen?

11.2 Why do you think this happens?

11.3 How do you react when your facility is running low on drugs for IPTp?

11.4 When you're running low on drugs for IPTp, does this affect how you administer IPTp to pregnant women attending antenatal care?

11.5 What do you do when the drug is not available at your facility?

11.6 How do you think stock-outs of drugs for IPTp could be avoided?

11.7 If you cannot offer IPTp to a woman who's attending antenatal care, do you suggest she obtain the tablets from elsewhere?

12. Sometimes, IPTp cannot be provided because there is no clean water or clean drinking cups are not available at the facility.

Has this ever happened at your facility?

12.1 If yes, how often does this happen?

12.2 How do you react when this happens?

12.3 What do you think could be done at your facility to ensure water and cups are available when needed?

13. Let's talk more about sulphadoxine/pyrimethamine, which you'll know as SP or Fansidar, the drug used for IPTp.

What do you know about this drug?

13.1 Do you have any concerns about how safe it is or how well it works as a drug?

13.2 In your experience, do women ever experience negative side effects after receiving IPTp?

13.3 If yes, what effects have you observed or heard of?

13.4 Weighing up what you know about IPTp and SP, how do you feel about the risks and benefits?

14. Are women required to pay for the provision of IPTp at your facility?

14.1 If yes, how much are they required to pay?

14.2 Have you ever experienced or heard of cases where women in public facilities were charged for services such as IPTp, the drugs or the materials, such as water and cups?

15. Finally, let's look at all the factors that affect how happy you are in your job.

Overall, how satisfied are you with the working conditions at your facility?

15.1 Why do you feel this way?

15.2 Does your workload affect whether you offer IPTp to pregnant women?

16. The Government of Uganda wants all eligible pregnant women to receive at least two doses of IPTp during the course of a pregnancy. Research shows, however, that fewer than half of all pregnant women do indeed receive the recommended two doses. In the course of this interview, we have discussed a number of the challenges with regard to providing and recording IPTp provision at the facility level.

Other than the challenges we have discussed, can you think of any additional issues that would need to be addressed in order to increase the number of pregnant women who receive two or more doses of IPTp as part of antenatal care?

17. In your opinion, what are the most important reasons why some women do not receive two or more doses of IPTp as part of antenatal care?

18. What are your main recommendations with regard to increasing the number of women in Uganda who receive the recommended two or more doses of IPTp as part of antenatal care?
