# Supplementary material for: Assessing supply-side barriers to uptake of intermittent preventive treatment for malaria in pregnancy: a qualitative study and document and record review in two regions of Uganda
Source: Malar J. 2016 Jul 4;15:341. doi: 10.1186/s12936-016-1405-4 (PMC4932731; doi:10.1186/s12936-016-1405-4)
Supplement: Supplementary file 3 — 10.1186/s12936-016-1405-4 Interview guide women (English). [file 12936_2016_1405_MOESM3_ESM.pdf]

Interview Guide – Women

**Interview Code**	
**Interviewee Code**	

**Name of Interviewer**	
**Date of Interview**	
**Time Started**	
**Time Finished**	
**Location**	
**Age of Interviewee**	
**Expected Month of Delivery**	
**Highest Educational Attainment**	

Introduction:

Good morning/ good afternoon

My name is [name]. I work as a researcher for Malaria Consortium, a not-for-profit organisation that specialises in the prevention, control and treatment of malaria and other diseases. We are currently carrying out research that will help us to find out how we can tailor our programmes so more people benefit from what we do. It was suggested by the village health team in your community that you might be a good person for us to speak to.

We have put together an information sheet that tells you more about the research we do, why you've been selected as a suitable interview partner and what happens if you agree to take part in the interview. Would you like to read it or would you prefer me or someone else to read it out for you?

[give time to read or read out information sheet]

Do you have any questions about this information? As the information sheet explains, you do not have to take part in the interview if you prefer not to.

If you agree to be interviewed, I will audio record the conversation and take notes throughout the interview. This will help me remember what you are telling me. Everything you say will be strictly confidential. Your replies will only be shared with the other researchers working on this study and will be identified by a number, not your name.

Can I please ask you to also read this consent form? I or someone else can read it out for you if you prefer.

[give time to read or read out consent form]

Do you have any questions about the information in this form? If you consent to being interviewed, can I please ask you to sign the form?

[interviewee and researcher sign consent form]

Thank you very much for agreeing to take part in this interview. I will now switch on the audio recorder. Remember that you do not have to answer questions you don't feel comfortable with and can end the interview at any time.

Let me begin by asking you a few questions about you and your pregnancy.

- What's your name?
- How old are you?
- When do you expect to deliver your baby?
- What is the highest grade you have completed in school?

1. I would like to talk with you about how you take care of yourself and your unborn baby when you're pregnant.

Can you tell me about how you make sure you and your baby are well when you're pregnant?

1.1 What do you do when you fall sick while you're pregnant?

2. Can you tell me about a time when you made a decision about health care or treatment while you were pregnant?

[Give interviewee time to think – if necessary prompt:]

For example, can you tell me about a time when you decided whether or not to visit a doctor or midwife or whether or not you should take medication while you were pregnant?

2.1 Do you seek guidance and advice from members of your family, household or community?

2.2 If there is disagreement about the right decision, who do you think should have the final say?

3. Let's talk more about a particular illness that can affect pregnant women and their unborn babies: malaria.

Do you think there is a difference between how malaria affects pregnant women and how it affects those who aren't pregnant?

3.1 What do you know about the risks of malaria to pregnant women and babies?

4. Do you know if there are any steps you can take to avoid getting malaria altogether?

4.1 What are they?

4.2 In your opinion, how well do they work?

4.3 Do you take any steps to avoid getting malaria?

5. Would you consider taking medication to avoid getting malaria while you're pregnant?

5.1 Why do you feel that way?

5.2 If you’re already taking steps to avoid getting malaria, do you think there is benefit in also taking medication?

6. The government recommends that pregnant women make a series of visits to a doctor or midwife during their pregnancy to check that mother and child are well, regardless of whether they feel sick or not. This service is called antenatal care.

Do you think it makes sense for pregnant women to attend this service?

6.1 Do you know how many check-up visits are recommended during the course of a pregnancy?

6.2 At what stages of the pregnancy are check-ups recommended?

7. During your pregnancy, how often have you gone for antenatal care so far?

7.1 At what stages of your pregnancy did those visits happen?

7.2 Did family or community members influence at all whether or not you went for antenatal care?

7.3 Did they influence how often you went for antenatal care?

8. Why did you decide to go for antenatal care when you did?

8.1 When you went for antenatal care for the first time during your pregnancy, what made you decide that this was the right time for a first visit?

9. Where did the visits take place?

9.1 Why did you decide to visit those facilities?

9.2 How far from those facilities do you live?

9.3 Can you describe how you travelled there?

9.4 Does the effort involved in travelling to the facility influence when and how often you visit a doctor or midwife for a check-up?

10. Thinking about a visit to a doctor or midwife for a check-up and taking everything into account, how much did you spend that day?

10.1 What did you spend it on?

10.2 Was it difficult for you to find the money to pay for the visit?

10.3 When deciding if and how often you visit a doctor or midwife for a check-up while you're pregnant, does the cost of these visits influence your decision?

10.4 Have you ever been asked by anyone working at a health facility to pay for any of the services or treatments you received when visiting a doctor or a midwife for a check-up?

10.5 How much were you asked to pay?

11. In your opinion, should pregnant women and their partners attend antenatal care together?

11.1 If yes, why do you think this would be a good idea?

11.2 If no, why not?

11.3 During your pregnancy, did your partner accompany you to an antenatal care visit?

11.4 If yes, how often did he accompany you?

12. Can you tell me about the services and treatments you received from your doctor or midwife when you went for antenatal care?

13. Overall, how satisfied were you with the treatment and the services you received?

13.1 What specifically was good or bad about the treatment and the service?

13.2 How could the service and the treatment have been better?

13.3 Did positive or negative experiences with the treatment and service you received from your doctor or midwife play a role in deciding whether or not to visit them for a check-up?

13.4 Did it affect how often you visited your doctor or midwife for a check-up?

14. Thinking about the doctors or midwives who treated you when you went for antenatal care, did you feel they were good at doing their job?

14.1 Did you feel they knew what they were doing?

14.2 In general, do you feel confident about the advice and recommendations you received from your doctor or midwife?

14.3 What do you think doctors and midwifes should do to make you feel confident about their advice and recommendations?

15. During antenatal care, pregnant women are often offered to take tablets which prevent them from getting malaria.

Have you heard of this medication?

15.1 What do you know about this medication?

15.2 Do you know the name of the drug?

16. When you attended antenatal care, did a doctor or a midwife offer you this medication?

16.1 If not offered, were you told why?

16.2 If not offered, did you get it from elsewhere?

16.3 When your doctor or midwife offered you the medication, did you agree to take it?

16.4 If no, why not?

16.5 At how many of your antenatal care visits were you offered the medication?

16.6 Did you take the tablets while the doctor or midwife were in the room with you?

16.7 If yes, were you provided with clean water and drinking cups to make swallowing the tablets safe and easy?

16.8 Were you concerned about how safe and clean the water and cups provided with the medication at the facility were?

16.9 If not offered under supervision, did you actually take the medication?

16.10 If not, why not?

17. Were you worried at all about the effects this drug might have on you or your baby?

17.1 What exactly were you worried about?

17.2 Did you experience any unpleasant side effects after taking the medication?

17.3 If yes, what kind of negative effects did you experience?

17.4 Have you heard about unpleasant experiences other women may have had with this drug?

18. Do you feel you have been given sufficient knowledge about this medication to allow you to make a decision with regard to whether or not to take it?

18.1 How do you think you should receive relevant information?

19. In the course of this interview, we have discussed a number of reasons why pregnant women may or may not take the medication that can protect them and their babies from the effects of malaria.

Overall, what would you say is the most important factor that determined whether or not to take the tablets?

20. The Government of Uganda wants all eligible pregnant women to receive the medication that protects them from malaria at least twice during the course of a pregnancy. However, at the moment, only about half of all pregnant women actually receive the medication twice.

If you could advise the government on what to do – what would you recommend should be done in order to increase the number of women who receive the medication?
