# Supplementary material for: Assessing supply-side barriers to uptake of intermittent preventive treatment for malaria in pregnancy: a qualitative study and document and record review in two regions of Uganda
Source: Malar J. 2016 Jul 4;15:341. doi: 10.1186/s12936-016-1405-4 (PMC4932731; doi:10.1186/s12936-016-1405-4)
Supplement: Supplementary file 4 — 10.1186/s12936-016-1405-4 Interview guide opinion leaders. [file 12936_2016_1405_MOESM4_ESM.pdf]

Interview Guide – Opinion Leaders

**Interview Code**	
**Interviewee Code**	

**Name of Interviewer**	
**Date of Interview**	
**Time Started**	
**Time Finished**	
**Location**	

Introduction:

Good morning/ good afternoon

My name is [name]. I work as a researcher for Malaria Consortium, a not-for-profit organisation that specialises in the prevention, control and treatment of malaria and other diseases. We are currently carrying out research that will help us to find out how we can tailor our programmes so more people benefit from what we do.

We have put together an information sheet that tells you more about the research we do, why you've been selected as a suitable interview partner and what happens if you agree to take part in the interview.

[give time to read information sheet]

Do you have any questions about this information? As the information sheet explains, you do not have to take part in the interview if you prefer not to.

If you agree to be interviewed, I will audio record the conversation and take notes throughout the interview. This will help me remember what you are telling me. Everything you say will be strictly confidential. Your replies will only be shared with the other researchers working on this study and you will be identified by a number, not your name. Can I please ask you to also read this consent form?

[give time to read consent form]

Do you have any questions about the information in this form? If you consent to being interviewed, can I please ask you to sign the form?

[interviewee and researcher sign consent form]

Thank you very much for agreeing to take part in this interview. I will now switch on the audio recorder. Remember that you do not have to answer questions you don't feel comfortable with and can end the interview at any time.

1. **I would like to talk with you about pregnant women and how they take care of their health.**

How do you think a pregnant woman should look after herself and her unborn baby?

1.1 In your community, do you advise pregnant women or their partners about their health?

1.2 What do you advise them on?

1.3 Are there any challenges in your community in terms of pregnant women's health?

1.4 What are the reasons for those challenges?

2. **The government recommends that pregnant women make a series of visits to a doctor or midwife during their pregnancy to check that mother and child are well, regardless of whether they feel sick or not. This service is called antenatal care.**

Do you think it makes sense for pregnant women to attend this service?

2.1 Why do you feel that way?

2.2 Do you know how many check-up visits are recommended during the course of a pregnancy?

2.3 Do you know if women in your community attend antenatal care?

3. **Do you know of any challenges that women from your community might experience in attending antenatal care?**

3.1 Why might women from your community not attend antenatal care?

3.2 Why might women not follow the recommended schedule of four antenatal care visits?

3.3 Why might women attend antenatal care late in their pregnancies?

3.4 Does the community influence whether women attend antenatal care?

4. **I would like to talk with you about one particular illness that can affect pregnant women and their unborn babies: malaria.**

Do you think there is a difference between how malaria affects pregnant women and how it affects those who aren't pregnant?

4.1 What do you know about the risks of malaria to pregnant women and babies?

5. **What do you think pregnant women should do to protect themselves and their unborn babies from malaria?**

5.1 Do you think pregnant women should take medication to prevent malaria?

5.2 Why do you feel that way?

6. **When women attend antenatal care, they are often offered to take tablets which prevent them from getting malaria. Have you heard of this medication?**

6.1 Do you know the name of the drug?

6.2 The medication is called IPTp or Fansidar. What do you know about this medication?

6.3 Do you know how often women should receive IPTp or Fansidar?

7. **Do you know if women from your community receive IPTp/Fansidar when they attend antenatal care?**

7.1 Do you know why women from your community might not receive IPTp/Fansidar?

7.2 Do you know if women ever refuse to take IPTp/Fansidar when offered by health workers?

7.3 Do you know why they might refuse to take IPTp/Fansidar?

7.4 Do you know whether the wider community influences whether women accept IPTp/Fansidar?

8. **The Government of Uganda wants all eligible pregnant women to receive IPTp/Fansidar at least twice during the course of a pregnancy. However, at the moment, only about half of all pregnant women actually receive the medication twice. In the course of this interview, we have discussed a number of reasons why pregnant women may or may not take the medication.**

If you could advise the government on what to do – what would you recommend should be done in order to increase the number of women who receive the medication?

8.1 What could be the role of the community in increasing uptake of IPTp/Fansidar?

8.2 How could the government and its partners support communities to increase uptake of IPTp/Fansidar?
